# Novel Series of Methyl 3-(Substituted Benzoyl)-7-Substituted-2-Phenylindolizine-1-Carboxylates as Promising Anti-Inflammatory Agents: Molecular Modeling Studies

**DOI:** 10.3390/biom9110661

**Published:** 2019-10-28

**Authors:** Katharigatta N. Venugopala, Omar H.A. Al-Attraqchi, Christophe Tratrat, Susanta K. Nayak, Mohamed A. Morsy, Bandar E. Aldhubiab, Mahesh Attimarad, Anroop B. Nair, Nagaraja Sreeharsha, Rashmi Venugopala, Michelyne Haroun, Meravanige B. Girish, Sandeep Chandrashekharappa, Osama I. Alwassil, Bharti Odhav

**Affiliations:** 1 Department of Pharmaceutical Sciences, College of Clinical Pharmacy, King Faisal University, Al-Ahsa 31982, Saudi Arabia; ctratrat@kfu.edu.sa (C.T.); momorsy@kfu.edu.sa (M.A.M.); baldhubiab@kfu.edu.sa (B.E.A.); mattimarad@kfu.edu.sa (M.A.); anair@kfu.edu.sa (A.B.N.); sharsha@kfu.edu.sa (N.S.); mharoun@kfu.edu.sa (M.H.); 2 Department of Biotechnology and Food Technology, Durban University of Technology, Durban 4001, South Africa; odhavb@dut.ac.za; 3 Faculty of Pharmacy, Philadelphia University, Amman 19392, Jordan; omar_attraq94@outlook.com; 4 Department of Chemistry, Visvesvaraya National Institute of Technology, Nagpur, Maharashtra 440010, India; sknayak@chm.vnit.ac.in; 5 Department of Pharmacology, Faculty of Medicine, Minia University, El-Minia 61511, Egypt; 6 Department of Public Health Medicine, University of KwaZulu-Natal, Howard College Campus, Durban 4001, South Africa; Venugopalar@ukzn.ac.za; 7 Department of Biomedical Sciences, College of Medicine, King Faisal University, Al-Ahsa 31982, Saudi Arabia; gmeravanige@kfu.edu.sa; 8 Institute for Stem Cell Biology and Regenerative Medicine, NCBS, TIFR, GKVK, Bellary Road, Bangalore 560065, India; 9 Department of Pharmaceutical Sciences, College of Pharmacy, King Saud bin Abdulaziz University for Health Sciences, Riyadh 11481, Saudi Arabia; wassilo@ksau-hs.edu.sa

**Keywords:** COX-2 inhibition, anti-inflammatory, indolizine derivatives, 2-phenyl indolizine, molecular modeling, absorption, distribution, metabolism, excretion, toxicity [ADMET] prediction

## Abstract

The cyclooxygenase-2 (COX-2) enzyme is considered to be an important target for developing novel anti-inflammatory agents. Selective COX-2 inhibitors offer the advantage of lower adverse effects that are commonly associated with non-selective COX inhibitors. In this work, a novel series of methyl 3-(substituted benzoyl)-7-substituted-2-phenylindolizine-1-carboxylates was synthesized and evaluated for COX-2 inhibitory activity. Compound **4e** was identified as the most active compound of the series with an IC_50_ of 6.71 μM, which is comparable to the IC_50_ of indomethacin, a marketed non-steroidal anti-inflammatory drug (NSAID). Molecular modeling and crystallographic studies were conducted to further characterize the compounds and gain better understanding of the binding interactions between the compounds and the residues at the active site of the COX-2 enzyme. The pharmacokinetic properties and potential toxic effects were predicted for all the synthesized compounds, which indicated good drug-like properties. Thus, these synthesized compounds can be considered as potential lead compounds for developing effective anti-inflammatory therapeutic agents.

## 1. Introduction

The non-steroidal anti-inflammatory drugs (NSAIDs) are considered to be one of the most frequently prescribed medications for the treatment of inflammation, pain, and fever [1]. The anti-inflammatory effects produced by the NSAIDs occur as a result of inhibition of the cyclooxygenase (COX) enzyme. This enzyme is involved in the synthesis of prostaglandin G2 (PGG2) from arachidonic acid, which is required for the inflammation process to take place [2,3]. There are mainly two important isoforms of the COX enzyme. The first one is the COX-1 isoform, which represents the constitute isoform that is distributed normally in certain tissues of the body such as the kidney and the gastrointestinal tract (GIT), this isoform carries out physiological functions such as gastric protection and maintenance of renal homeostasis [4,5,6]. The other isoform is the COX-2, which is the inducible isoform that is only expressed during exposure to certain substances such as cytokines, which are produced during injury. The use of non-selective COX inhibitors, which inhibit both the COX-1 and COX-2 isoforms usually, results in undesirable adverse effects, mainly involving the GIT. These adverse effects are caused by the inhibition of the COX-1 isoform, which is responsible for GIT protection. For instance, GIT ulceration is a common side effect of non-selective NSAIDs, which results mainly from the inhibition of the COX-1 isoform [7,8]. In order to develop anti-inflammatory agents that have an improved safety profile and reduced side effects, the discovery of selective COX-2 inhibitors is required, as these inhibitors will be devoid of the side effects observed from COX-1 isoform inhibition.

**Figure 1:**
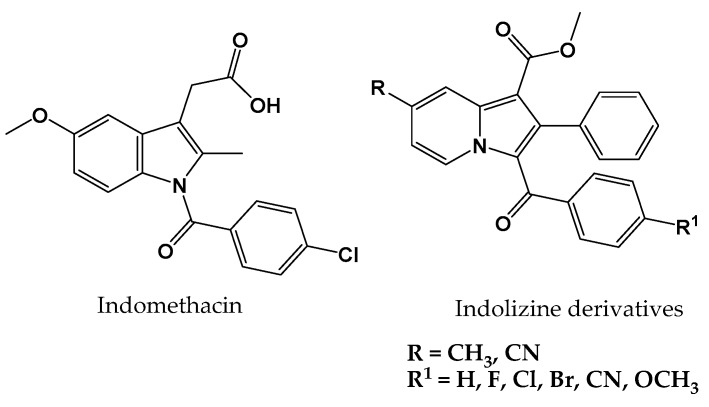
Commercially available COX-2 enzyme inhibitor (indomethacin) and the proposed methyl 3-(substituted benzoyl)-7-substituted-2-phenylindolizine-1-carboxylate analogues for COX-2 inhibition activity.

Compounds based on the indolizine scaffold (Figure 1) have been reported to possess a wide spectrum of biological activities. For instance, they have been shown to be able to bind to phospholipase A2, histamine receptors, and calcium channels, which are well-known drug targets [9,10]. In addition, indolizine derivatives were reported to have potential anti-inflammatory and anti-cancer effects [11,12,13]. Figure 1 shows the similar structures of the marketed COX-2 inhibitor indomethacin and the scaffold of the indolizine derivatives.

In continuation of our effort to identify potential COX-2 inhibitors [14,15], in the present study, the anti-inflammatory activity of a series of phenylindolizine derivatives was evaluated by assessing the ability of the compounds to inhibit the COX-2 enzyme. In addition, molecular modeling studies were carried out to gain better insights into the structural requirements for achieving the high activity. The pharmacokinetic properties and toxicity of the synthesized compounds were predicted to evaluate their potential as lead compounds for developing anti-inflammatory agents.

## 2. Materials and Methods

### 2.1. General

All chemicals reported here were obtained from Sigma-Aldrich Co. (St. Louis, MO, USA), while the solvents were obtained from MilliporeSigma (Burlington, MA, USA). Thin-layer chromatography (TLC) was employed to observe chemical reactions, and this process was performed on silica gel (Sigma-Aldrich Co., St. Louis (HQ), MO, USA), on aluminum foil; *n*-hexane and ethyl acetate (4:6) were used as the solvent. The reactions were visualized under an ultraviolet (UV)-light/iodine chamber. A Büchi melting point B-545 apparatus was used to measure the melting points (Büchi, Labortechnik, Flawil, Switzerland). Infrared (IR) spectra were recorded on a Shimadzu IRAffinity-1S Fourier-transform infrared (FT-IR) spectrometer (Shimadzu Corporation, 1 Nishinokyo Kuwabara-cho, Nakagyo-ku, Kyoto 604-8511, Japan. Further, ^1^H and ^13^C-nuclear magnetic resonance (NMR) spectra were recorded using Bruker AVANCE III 400 MHz (Bruker Corporation, Billerica, MA, USA) with CDCl_3_ (solvent). Chemical shifts (*δ*) were indicated in ppm, with tetramethylsilane (TMS) as a reference; coupling constants (J) were recorded (Hz). The splitting pattern was documented as follows: s, singlet; d, doublet; q, quartet; and m, multiplet. Liquid chromatography–mass spectrometry (LC-MS) (Agilent 1100 series, (Agilent Technologies Agilent Technologies, 5301 Stevens Creek Blvd, Santa Clara, CA, USA) was used to measure the mass spectra, in conjunction with MSD and 0.1% aqueous trifluoroacetic acid in an acetonitrile system on the C18-BDS column. Elemental analysis was conducted using a FLASH EA 1112 CHN analyzer (Thermo Finnigan LLC, New York, NY, USA).

### 2.2. General Procedure for the Synthesis of Methyl 3-(Substituted Benzoyl)-7-Substituted-2-Phenylindolizine-1-Carboxylate Analogues (***4a***–***g***)

**Scheme 1:**
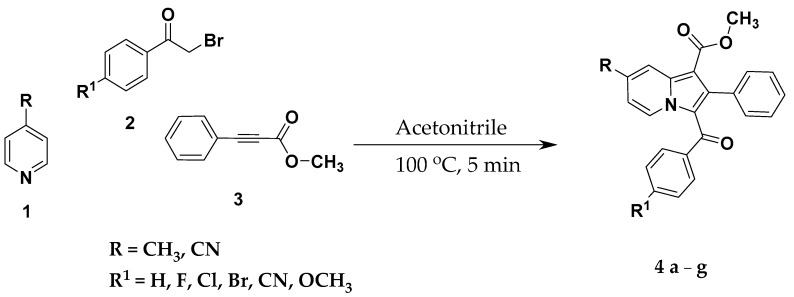
Synthesis of methyl 3-(substituted benzoyl)-7-substituted-2-phenylindolizine-1-carboxylate analogues **4a**–**g**. FT-IR and ^1^H-NMR of **4a**–**g** see Supplementary Materials.

As indicated in Scheme 1, a mixture of 4-methyl/cyano pyridine (1.00 mmol), 4-substituedphenacyl bromide (1.00 mmol), and methyl phenylpropiolate (1.00 mmol) was added to 4 mL acetonitrile and triethylamine (1.00 mmol) in 10 mL of microwave tube under nitrogen atmosphere. The reaction mixture was irradiated at 100 °C in a microwave initiator up to 5 min. The reaction completion was monitored on TLC. After reaction completion, the reaction medium was evaporated under reduced pressure, the crude product obtained was diluted with water, the aqueous layer was extracted twice with ethyl acetate and the organic layer was washed with brine solution. The organic layer was evaporated under reduced pressure, and the product obtained was purified by column chromatography using 60–120 mesh silica gel with hexane and ethyl acetate solvent system to afford 76%–89% yield of methyl 3-(substituted benzoyl)-7-substituted-2-phenylindolizine-1-carboxylates.

*Methyl 3-Benzoyl-7-Methyl-2-Phenylindolizine-1-Carboxylate* (**4a**). Appearance: yellow crystalline compound, Yield: 87%, melting point: 173–174 °C, FT-IR (KBr, cm^−1^): 2945.10, 1708.81 (C=O), 1591.16 (C=O), 1444.58, 1413.72, 1217.00, 1168.78, 1080.06, 821.62, 703.97, 563.18; ^1^H-NMR (400 MHz) *δ* = 9.56 (d, *J* = 7.2 Hz, 1H), 8.25 (s, 1H), 7.36–7.34 (d, *J* = 7.6 Hz, 2H), 7.16–7.09 (m, 3H), 7.03–7.00 (m, 5H), 6.92–6.90 (m, 1H), 3.70 (s, 3H), 2.53 (s, 3H); ^13^C-NMR (100 MHz) *δ* = 188.13, 164.95, 140.77, 139.68, 139.49, 138.87, 133.94, 131.44, 131.15, 130.82, 129.10, 127.65, 127.35, 127.08, 126.75, 122.08, 118.44, 117.39, 103.42, 50.77, 21.64; LC-MS (ESI, Positive): *m*/*z*: [M + H]^+^: 370; Anal. calculated for: C_24_H_19_NO_3_; C, 78.03; H, 5.18; N, 3.79; Found: C, 78.07; H, 5.19; N, 3.89.

*Methyl 3-(4-Fluorobenzoyl)-7-Methyl-2-Phenylindolizine-1-Carboxylate* (**4b**). Appearance: yellow crystalline compound, Yield: 85%, melting point: 167–168 °C, FT-IR (KBr, cm^−1^): 2972.10, 1685.67 (C=O), 1602.74 (C=O), 1379.01, 1217.00, 1118.64, 867.91, 783.05, 605.61, 522.67; ^1^H-NMR (400 MHz) *δ* = 9.53 (d, *J* = 7.2 Hz, 1H), 8.25 (s, 1H), 7.37–7.34 (m, 2H), 7.10–7.02 (m, 5H), 6.92–6.91 (d, *J* = 7.2Hz, 1H), 6.70–6.66 (t, *J* = 8.4Hz, 2H), 3.68 (s, 3H), 2.55 (s, 3H); ^13^C-NMR (100 MHz) *δ* = 186.54, 165.33, 164.88, 162.83, 140.71, 139.76, 139.03, 135.72, 135.69, 133.86, 131.52, 131.34, 131.16, 127.61, 127.25, 126.89, 121.87, 118.49, 117.48, 114.53, 114.31, 103.48, 50.81, 21.64; LC-MS (ESI, Positive): m/z: [M + H]^+^: 388; Anal. calculated for: C_24_H_18_FNO_3_; C, 74.41; H, 4.68; N, 3.62; Found: C, 74.43; H, 4.67; N, 3.63.

*Methyl 3-(4-Chlorobenzoyl)-7-Methyl-2-Phenylindolizine-1-Carboxylate* (**4c**). Appearance: yellow crystalline compound, Yield: 89%, melting point: 154–155 °C, FT-IR (KBr, cm^−1^): 2945.10, 1708.81 (C=O), 1593.09 (C=O), 1415.65, 1290.29, 1217.00, 918.05, 821.62, 707.83, 563.18; ^1^H-NMR (400 MHz) *δ* = 9.58 (d, *J* = 7.2 Hz, 1H), 8.26 (s, 1H), 7.26–7.24 (d, *J* = 8.0 Hz, 2H), 7.10–7.02 (m, 5H), 6.98–6.92 (m, 3H), 3.70 (s, 3H), 2.54 (s, 3H); ^13^C-NMR (100 MHz) *δ* = 186.63, 164.83, 141.02, 139.84, 139.24, 137.94, 136.78, 133.80, 131.13, 130.36, 127.71, 127.57, 127.20, 126.93, 121.83, 118.51, 117.59, 103.65, 50.82, 21.65; LC-MS (ESI, Positive): *m*/*z*: ([M + H]^+^: 404; Anal. calculated for: C_24_H_18_ClNO_3_; C, 71.38; H, 4.49; N, 3.47; Found: C, 71.33; H, 4.45; N, 3.52.

*Methyl 3-(4-Bromobenzoyl)-7-Methyl-2-Phenylindolizine-1-Carboxylate* (**4d**). Appearance: brown crystalline compound, Yield: 85%, melting point: 137–138 °C, FT-IR (KBr, cm^−1^): 2975.96, 1689.53 (C=O), 1614.31 (C=O), 1421.44, 1217.00, 1178.43, 1108.99, 798.47, 757.97, 636.47, 595.96; ^1^H-NMR (400 MHz) *δ* = 9.59 (d, *J* = 7.2 Hz, 1H), 8.25 (s, 1H), 7.18–7.16 (m, 2H), 7.13–7.02 (m, 7H), 6.94–6.92 (d, *J* = 7.2 Hz, 1H), 3.69 (s, 3H), 2.53 (s, 3H); ^13^C-NMR (100 MHz) *δ* = 186.72, 164.82, 141.10, 139.85, 139.28, 138.39, 133.78, 131.12, 130.53, 130.46, 127.74, 127.17, 126.95, 125.33, 121.80, 118.50, 117.62, 103.70, 50.82, 21.66; LC-MS (ESI, Positive): *m*/*z*: [M + H] ^+^: 448; Anal. calculated for: C_24_H_18_BrNO_3_; C, 64.30; H, 4.05; N, 3.12; Found: C, 64.35; H, 4.06; N, 3.06.

*Methyl 7-Cyano-3-(4-Cyanobenzoyl)-2-Phenylindolizine-1-Carboxylate* (**4e**). Appearance: yellow amorphous compound, Yield: 76%, melting point: 235–236 °C, FT-IR (KBr, cm^−1^): 2950, 2223 (CN), 1718 (C=O), 1627 (C=O), 1375, 1226.64, 1080.06, 925.77, 702.04, 549.67; ^1^H-NMR (400 MHz) *δ* = 9.55 (d *J* = 7.2 Hz, 1H), 8.02 (s, 1H), 7.86–7.84 (d, *J* = 6.54 Hz, 2H), 7.79–7.77 (d, *J* = 6.54 Hz, 2H), 7.53–7.31 (m, 5H), 7.10–7.05 (m, 1H), 3.76 (s, 3H); ^13^C-NMR (400 MHz) *δ* = 184.77, 164.30, 143.63, 133.21, 132.24, 131.35, 131.09, 130.49, 129.89, 129.28, 128.99, 128.60, 127.83, 127.38, 125.50, 121.83, 121.70, 117.83, 115.51, 114.96,107.36, 52.05; LC-MS (ESI, Positive): *m*/*z*: [M + H] ^+^: 406; Anal calculated for: C_25_H_15_N_3_O_3_: C, 74.07, H, 3.73, N, 10.36: Found: C, 73.99, H, 3.68, N, 10.42.

*Methyl 3-(4-Bromobenzoyl)-7-Cyano-2-Phenylindolizine-1-Carboxylate* (**4f**). Appearance: brown crystalline compound, Yield: 84%, melting point: 208–209 °C, FT-IR (KBr, cm^−1^): 2950.89, 2225.70 (CN), 1706.88 (C=O), 1608.52 (C=O), 1585.36, 1514.02, 1440.51, 1419.51, 1217.00, 1143.71, 1072.35, 918.05, 705.90, 543.89; ^1^H-NMR (400 MHz) *δ* = 9.49–9.48 (m, 1H), 8.84 (s, 1H), 7.44–7.42 (d, *J* = 7.2 Hz, 2H), 7.23–7.13 (m, 6H), 7.12–7.08 (m, 4H), 3.77 (s, 3H); ^13^C-NMR (400 MHz) *δ* = 187.29, 163.70, 140.61, 137.01, 136.29, 132.33, 131.81, 131.10, 130.90, 129.99, 128.42, 128.14, 127.93, 127.32, 126.79, 119.90, 117.35, 115.13, 114.42, 109.35, 107.39, 51.46; LC-MS (ESI, Positive): *m*/*z*: [M + H] ^+^: 459.2; Anal calculated for: C_24_H_15_BrN_2_O_3_: C, 62.76, H, 3.29, N, 6.10: Found: C, 62.75, H, 3.26, N, 6.12.

*Methyl 7-Cyano-3-(4-Methoxybenzoyl)-2-Phenylindolizine-1-Carboxylate* (**4g**). Appearance: yellow crystalline compound, Yield: 81%, melting point: 166–167 °C, FT-IR (KBr, cm^−1^): 2950.89, 2227.63, 1706.88 (C=O), 1600.81 (C=O), 1573.81, 1514.02, 1369.37, 1220.86, 1141.78, 1027.99, 802.33, 709.76, 615.25; ^1^H-NMR (400 MHz) *δ* = 9.21–9.20 (m, 1H), 8.81 (s, 1H), 7.44–7.42 (d, *J* = 7.2 Hz, 2H), 7.17–7.15 (m, 3H), 7.10–7.05 (m, 3H), 6.57–6.55 (d, *J* = 7.2 Hz, 2H), 3.78 (s, 3H), 3.72 (s, 3H); ^13^C-NMR (400 MHz) *δ* = 186.95, 163.92, 162.95, 139.14, 135.72, 132.61, 131.88, 131.14, 130.50, 130.13, 128.68, 127.75, 127.25, 126.37, 124.44, 117.63, 113.88, 113.77, 113.13, 108.34, 106.86, 55.56, 51.96; LC-MS (ESI, Positive): *m*/*z*: [M + H] ^+^: 411; Anal calculated for: C_25_H_18_N_2_O_4_: C, 73.16, H, 4.42, N, 6.83: Found: C, 73.08, H, 4.38, N, 6.90.

### 2.3. Crystallography

Single crystal of compound **4a** was obtained from mixture of acetone and ethyl alcohol solvent at 1:1 ratio by slow evaporation method at low temperature as block shape and brown in color. Single-crystal X-ray diffraction data were collected on a Bruker KAPPA APEX II DUO diffractometer using graphite-monochromated Mo-Kα radiation (χ = 0.71073 Å). Data collection was carried out at 173(2) K. Temperature was controlled by an Oxford Cryostream cooling system (Oxford Cryostat). Cell refinement and data reduction were performed using the program SAINT [16]. The data were scaled and absorption correction performed using SADABS [17]. The structure was solved by direct methods using SHELXS-97 [17] and refined by full-matrix least-squares methods based on F^2^ using SHELXL-2014 [17] and using the graphics interface program X-Seed [18,19]. Both the programs X-Seed and POV-Ray [20] were used to prepare molecular graphic images. All non-hydrogen atoms were refined anisotropically. All hydrogen atoms were placed in idealized positions and refined in riding models with Uiso assigned 1.2 or 1.5 times Ueq of their parent atoms and the C–H bond distances were constrained in the range from 0.95 Å to 0.99 Å. The structure was refined to R factor of 0.0462.

### 2.4. Pharmacology

#### 2.4.1. COX-2 Inhibition Assay

In vitro, a COX-2 inhibition study of the designed methyl 3-(substituted benzoyl)-7-substituted-2-phenylindolizine-1-carboxylate analogues (**4a**–**g**) was conducted; they were screened for in vitro human recombinant COX-2 enzyme inhibitory activity as described previously [14].

#### 2.4.2. Statistical Analysis

The one-way investigation of variance (ANOVA) was used to compare the in vitro COX 2 inhibitory activity of the designed the methyl 3-(substituted benzoyl)-7-substituted-2-phenylindolizine-1-carboxylate analogues (**4a**–**g**) with nonselective (indomethacin) and selective (celecoxib) standard substances. The steps were followed as we described in our previous communication [14].

### 2.5. Computational Studies

#### 2.5.1. Molecular Docking

Computational modeling studies were conducted using Accelry’s Discovery Studio 4.0 client program, in which the algorithm used was CHARMm force fields. The X-ray co-crystal of COX-2 enzyme and indomethacin (PDB: 4COX) was used to estimate the binding mode of indolizines. The molecular interaction of indolizines and the enzyme was determined by following steps from our recently reported docking protocol [14].

#### 2.5.2. ADME Prediction

In this study, the pharmacokinetic properties of all the synthesized compounds were predicted and analyzed. The SwissADME webserver was used for calculating and predicting various parameters related to the ADME properties of the compounds [21]. In particular, parameters related to the oral absorption property such as the number of rotatable bonds, the octanol/water partition coefficient (Log*P*), and the molecular weight were calculated. The ability of the compounds to cross the blood–brain barrier (BBB) was predicted as compounds entering the central nervous system (CNS) might have undesirable adverse effects that need proper considerations [22]. In addition, the probability of binding to the p-glycoprotein (P-gp) was predicted. Since this protein is known to act as an efflux pump to many xenobiotics, compounds that are substrate to this protein might be prone to resistance, hence structural modifications might be required to prevent binding to P-gp [23,24].

#### 2.5.3. Toxicity Prediction

In this study, the SwissADME web server and the DataWarrior software were used to predict several toxic effects of the synthesized compounds [21,25]. The potential inhibition of some important cytochrome P450 (CYP 450) isoforms by the compounds was predicted. Inhibition of CYP 450 isoforms needs to be taken into consideration since it may alter the metabolism of the other drugs causing drug–drug interactions [26]. In addition, to further characterize the toxicity profiles of the synthesized compounds, the mutagenicity, tumorigenicity, and reproductive toxicity were predicted for all the compounds.

## 3. Results and Discussion

### 3.1. Chemistry

The synthetic route for the construction of target compounds, methyl 3-(substituted benzoyl)-7-substituted-2-phenylindolizine-1-carboxylate analogues (**4a**–**g**) is illustrated in Scheme 1. The treatment of 4-substituted pyridine, 4-substituedphenacyl bromide, and methyl phenylpropiolate in presence of triethylamine and acetonitrile yielded the title compounds **4a**–**g** at 76%–89% yield by one-pot microwave-assisted synthetic approach. The purity of the compounds was confirmed by HPLC, and it was found to be over 99%. The physicochemical characteristics of methyl 3-(substituted benzoyl)-7-substituted-2-phenylindolizine-1-carboxylate analogues **4a**–**g** are tabulated in Table 1. The chemical structure of the products **4a**–**g** was elucidated by FT-IR, NMR (^1^H and ^13^C), mass spectrometry, and elemental analysis. The FT-IR spectra of the synthesized compounds exhibited stretching absorption band in the range of 1685–1718 cm^−1^ for carbonyl group (C=O). The characteristic cyano absorption peak for compounds **4e**–**g** is found in the range of 2223–2227 cm^−1^. For compounds **4d** and **4f** carbon–bromine stretching vibration is observed in the range of 626.82–636.47 cm^−1^. For compounds **4c** and **4b,** carbon–chlorine and carbon–fluorine stretching vibrations are observed at 707.85 and 1217.00 cm^−1^, respectively. ^1^H-NMR spectra revealed the appearance of a singlet for a methyl group at the seventh position of the indolizine nucleus (**4a**–**d**) and a singlet for an ester methyl group at the first position of the indolizine nucleus (**4e**–**g**) are observed in the ranges of 3.68–3.78 and 2.53–2.55 ppm, respectively. In case of para-methoxy derivative **4g**, a singlet for methoxy is observed at 3.72 ppm. The doublet peaks were commonly seen in all the derivatives **2a**–**g** with *J* value at 7.12 Hz as para substituents such as fluoro, chloro, bromo, methoxy, and cyano on phenyl ring at the third position of the indolizine nucleus. In case of title compound **4b**, the triplet peak was observed in the range of 6.70–6.66 ppm with *J* value at 8.4 Hz. The ^13^C-NMR spectra revealed the appearance of carbonyl carbon for compounds **4a**–**g** in the range of 184.77–187.29 ppm. The title compounds **4a**–**g** revealed ester alkyl carbon, which is at first position of the indolizine nucleus is found in the range of 50.77–52.05 ppm. The title compounds **4e**–**d** exhibited a peak for a methyl group, which is at seventh position of the indolizine nucleus in the range of 21.64–21.66 ppm. The title compound **4g** revealed a peak at 55.56 ppm due to the methoxy group at para position of benzoyl group, which is at third position of the indolizine nucleus. In LC-MS spectra of the compounds **4a**–**g** the molecular ion peaks were in good agreement with molecular mass of the compounds. The results of elemental analysis were in good agreement with the calculated values of the proposed tile compounds **4a**–**g**. The cLog*P* of the proposed tile compounds **4a**–**g** was calculated using ChemDraw Professional 16.0 (PerkinElmer Informatics, Inc. 940 Winter Street Waltham, MA, USA) and the results were in the range of 4.6463–7.0802.

### 3.2. Crystallography

The parameters for crystal data collection and structure refinements, the bond lengths, angles, and torsion angles are contained in CIF file which was deposited in the Cambridge Crystallographic Data Centre (CCDC) [27] with CCDC number 1950516. The crystallographic details are listed in Table 2. Intermolecular interactions, thermal ellipsoid diagram, and packing diagrams were generated using CSD software ORTEP [28] and Mercury 3.8 [29].

**Figure 2:**
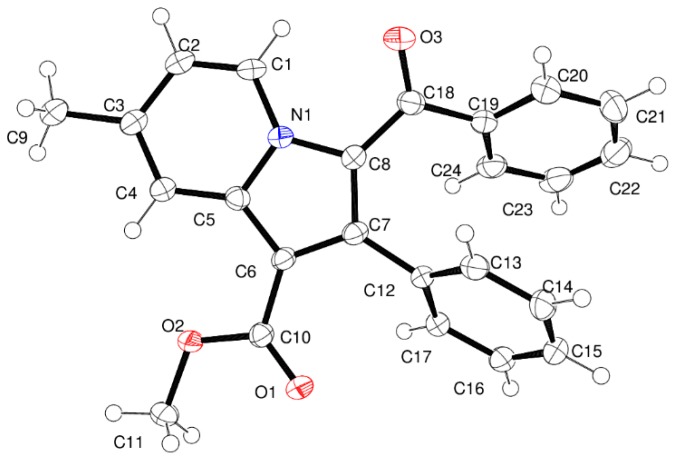
The asymmetric unit of compound **4a** with 50% probability thermal ellipsoids.

**Figure 3:**
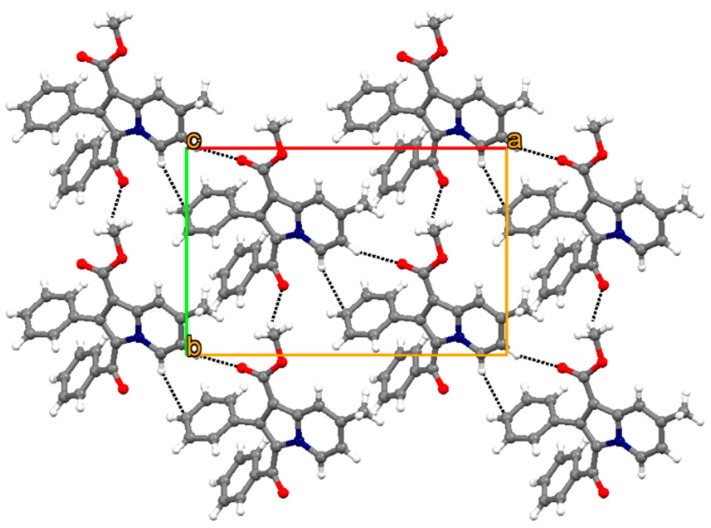
Molecular assembly of **4a** shown along [1 1 0] plane through weak C–H···O and C–H···π hydrogen bondings.

Figure 2 shows the thermal ellipsoid plot of **4a** with atom labelling. Crystal structure of **4a** forms molecular sheet assembly through weak C–H∙∙∙O and C–H∙∙∙π hydrogen bonds (Table 3 and Figure 3). The remaining molecules of this series of phenylindolizine are expected to have the same molecular assembly as their conformations have the same molecular core moiety and functional groups.

### 3.3. Pharmacology

#### COX-2 Inhibition

A series of methyl 3-(substituted benzoyl)-7-substituted-2-phenylindolizine-1-carboxylate analogues **4a**–**g** were evaluated for in-vitro COX-2 inhibition activity and it was found that all the compounds displayed inhibitory effects at IC_50_ values between 6.71–41.59 (Table 4). From the series, compound **4e** emerged as the most promising COX-2 inhibitor with a cLog*P* value of 4.6463 and having two cyano groups on indolizine and benzoyl moiety with IC_50_ of 6.71. However, compound **4g** exhibited moderate inhibitory activity at IC_50_ 9.62, having a cyano group on the indolizine ring and methoxy at the para position of the benzoyl ring, when compared to indomethacin standard. In the case of compounds **4a**, **4b**, **4c,** and **4d,** methyl functional group on the indolizine nucleus did not favor promising COX-2 inhibitory activity. The COX-2 inhibitory activity of the test compounds (**4a**–**4d**) having a methyl group at seventh position of the indolizine nucleus was in the order **4b** > **4d** > **4c** > **4a**. The COX-2 inhibitory activity of the compounds (**4e**–**g**) having a cyano group at seventh position of the indolizine nucleus was in the order **4e** > **4g** > **4f**.

### 3.4. Computational Studies

#### 3.4.1. Molecular Modeling

**Figure 4:**
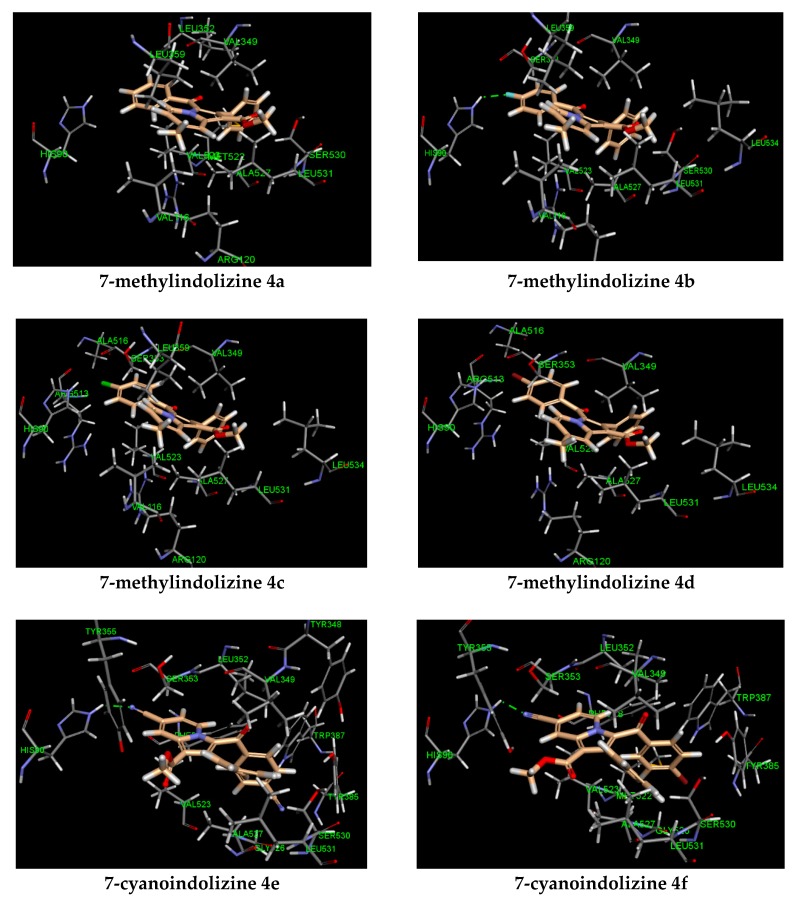

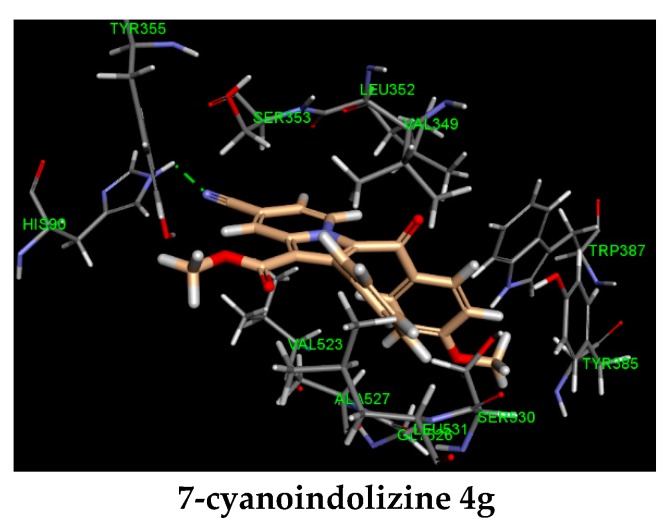
Predicted docking pose of indolizines **4b**–**g** (salmon-filled spheres) in the COX-2 domain (PDB 4COX). Hydrogen bonding interaction is represented as a green dotted line.

Two novel 2-phenylindolizine series, 7-methyl, and 7-cyano were investigated for COX-2 inhibitory activity. The bioactivity study revealed that 7-cyanoindolines were more active than their congener 7-methylindolizines. To gain insights into the potency of the synthesized indolizines, the key interactions between the compounds and COX-2 active site were examined through molecular docking study and reported in Table 4. Molecular modeling analysis showed two distinct binding modes for each series (Figure 4). The indolizino ring of 7-cyano series was pointed toward the residue HIS 90 participating in hydrogen bonding with cyano group at position 7 (compounds **4e**–**g**, Figure 4), whereas the indolizino ring of 7-methyl series was projected toward the amino acid ARG 120 by interacting with the indolizino ring through pi–cation interaction (compounds **4b**–**d**, Figure 4). The different conformation in binding site between the two series may explain the observed COX-2 activity. In the 7-methyl series, the hydrophobic interactions were mainly observed between the residues VAL 116, LEU 359, LEU 531 and methyl at position 7, and the amino acids HIS 90, ARG 513, ALA 516 and chlorine (**4c**) and bromine (**4d**) at the benzoyl ring with the exception of 3-(4-fluorobenzoyl) indolizine **4b** (Figure 4). The latter exhibited strong hydrogen bonding with the residue HIS 90 and fluorine. The 7-cyanoindolizines **4e**–**g** displayed strong H-bond with the residue HIS 90 and the cyano group at position 7 conferring greater inhibitory activity over the 7-methylindolizines **4a**–**d**. Another interesting binding feature between the two series is the involvement of the amino acid ARG 120 in pi–cation interaction observed for only the 7-methylindolizine series. The lack of selectivity between COX-2 and COX-1 inhibitors is due to the ion pair or/and hydrogen bonding interaction with ARG 120 [30,31,32]. The non-involvement of such interaction with the 7-cyanoindolizines **4e**–**g** may exert greater selectivity over 7-methylindolizines **4a**–**d**.

#### 3.4.2. ADME Prediction

The prediction of the pharmacokinetic properties of lead compounds at an early stage of the drug design and development process can greatly assist in the proper selection of compounds for further development [33]. In fact, a relatively high number of compounds fail to make it to the market because of pharmacokinetic related issues that appear in later stages of the drug design and development process [33,34]. The results of the pharmacokinetic parameters prediction are listed in Table 5. In general, most of the compounds were predicted to have favorable pharmacokinetic properties. In terms of oral absorption, all the compounds fulfill the conditions of Lipinski’s rule of five criteria. The number of rotatable bonds for all the compounds is less than seven rotatable bonds, indicating a high probability of being orally bioavailable [35]. Also, The GI absorption was predicted to be high for all the compounds. Compounds **4a, 4b, 4c,** and **4d** were predicted to be BBB permeant, which means they have the ability to cross the BBB and enter the CNS while the remaining compounds were predicted to lack the ability to cross the BBB. All the compounds were predicted to have low probability of being P-gp substrates, which means there is low possibility of developing resistance by P-gp efflux.

#### 3.4.3. Toxicity Prediction

The prediction of potential toxic effects of the compounds at an early stage of the drug design and development process is important in a similar manner to ADME prediction. The results of toxicity prediction are shown in Table 6. In general, the compounds were predicted to have a good safety profile. In terms of mutagenicity, tumorigenicity, and reproductive toxicity, all the compounds were predicted to be devoid of such major toxic effects. Also, none of the compounds was predicted to have irritant effects. In terms of CYP 450 isoforms inhibition, all the compounds except compound **4f** were predicted to be potential inhibitors of the CYP1A2 isoform. On the other hand, none of the compounds was predicted to be a potential inhibitor of the CYP2D6 isoform. Compounds **4e** and **4g** were predicted to be CYP3A4 inhibitors. Thus, the compounds appear to have the ability to inhibit the CYP1A2 isoform, which requires consideration during further development of the compounds to avoid any possible drug–drug interactions resulting from CYP1A2 inhibition [26].

## 4. Conclusions

In this study, a series of a novel series of methyl 3-(substituted benzoyl)-7-substituted-2-phenylindolizine-1-carboxylates was synthesized and assessed for COX-2 inhibition activity. All the compounds exhibited moderate to good activity as COX-2 inhibitors. The most active compound was compound **4e**, which has an IC_50_ of 6.71 μM, similar to the IC_50_ of the marketed NSAID indomethacin. Molecular docking was conducted to understand the binding interactions taking place between the compounds and the amino acid residues in the active site of the COX-2 enzyme. The ADME properties, as well as the potential toxic effects of the synthesized compounds, were predicted. The results indicated a high probability for oral bioavailability for all the compounds. Also, none of the compounds was predicted to have any major toxic effects. Thus, these novel COX-2 inhibitors have the potential to be used as lead compounds for the development of improved COX-2 inhibitors.

## Figures and Tables

**Table 1:** Physicochemical characteristics of methyl 3-(substituted benzoyl)-7-substituted-2-phenylindolizine-1-carboxylate analogues **4a**–**g**.

Compound Code	Mol Formula (Mol Weight)	R	R^1^	Yield (%) ^a^	M.P. (°C)	CLogP ^b^
**4a**	C_24_H_19_NO_3_ (369)	CH_3_	H	87	173–174	6.2094
**4b**	C_24_H_18_FNO_3_ (387)	CH_3_	F	85	167–168	6.3602
**4c**	C_24_H_18_ClNO_3_ (403)	CH_3_	Cl	89	154–155	6.9302
**4d**	C_24_H_18_BrNO_3_ (447)	CH_3_	Br	85	137–138	7.0802
**4e**	C_25_H_15_N_3_O_3_ (405)	CN	CN	76	235–236	4.6463
**4f**	C_24_H_15_BrN_2_O_3_ (458)	CN	Br	84	208–209	6.0300
**4g**	C_25_H_18_N_2_O_4_ (410)	CN	OCH_3_	81	166–167	5.3301

^a^ Yields calculated after being purified using the recrystallization method; ethanol was used as the solvent. ^b^ cLog*P* of the compounds was calculated using ChemDraw Professional 16.

**Table 2:** Single crystal data collection and refinement for title compounds **4a**.

DATA	Compound 4a
Formula	C_24_H_19_ N O_3_
Formula weight	369.40
Temperature/K	153 (2)
Wavelength (Å)	0.71073
Crystal system	monoclinic
Space group	*C 2/c*
*a* (Å)	16.550 (4)
*b* (Å)	10.391 (2)
*c* (Å)	22.179 (4)
*α* (°)	90
*β* (°)	104.468 (4)
*γ* (°)	90
V (Å^3^)	3693.1 (13)
Z’, Z	1, 8
Density (g cm^−1^)	1.329
μ (mm^−1^)	0.088
F (000)	1552
θ (min, max)	2.336, 25.413
h_min, max_; k_min, max_; l_min, max._	−19, 19; −12, 12; −26, 26
No. of refl.	3375
No of unique ref./Obs. ref.	3375, 2340
No. parameters	255
R_all,_ R_obs_	0.0462, 0.0815
wR_all,_ wR_obs_	0.113, 0.099
Δρ_min_, _max_ (eÅ^−3^)	−0.272, 0.195
G.O.F.	1.020

**Table 3:** Intermolecular interactions of compounds **4a**.

D–X···A	D–X (Å)	X···A (Å)	D···A (Å)	<D–X···A (Å)	Symmetry Code
C2–H2···O1	0.95	2.56	3.450 (3)	156	1/2 + x, 1/2 + y, z
C11–H11A···O3	0.98	2.53	3.383 (3)	146	x, −1 + y, z
C17–H17···Cg	0.98	2.83	3.678	149	1/2 − x, −1/2 + y, 1/2 − z

Cg = the centroid of six-membered ring C19/C20/C21/C22/C23/C24.

**Table 4:** In vitro COX-2 inhibitory activity and docking results of methyl 3-(substituted benzoyl)-7-substituted-2-phenylindolizine-1-carboxylate scaffolds **4a**–**g**.

Entry	R_1_	R_2_	IC_50_ (μM)^a^	CDocker E. (kcal/mol)	Hydrogen Bonding (Interacting Atom, Å)	Pi Interaction
**4a**	CH_3_	H	41.59 ± 0.03 ^a^	−31		ARG 120 (pi–cation)
**4b**	CH_3_	F	27.08 ± 0.03 ^c,d^	−34	HIS 90 (F, 2.09)	ARG 120 (pi–cation)
**4c**	CH_3_	Cl	38.11 ± 0.03 ^b,d^	−33		ARG 120 (pi–cation)
**4d**	CH_3_	Br	37.66 ± 0.03 ^d^	−32		ARG 120 (pi–cation)
**4e**	CN	CN	6.71 ± 0.03 ^b^	−35	HIS 90 (CN, 2.02)	
**4f**	CN	Br	13.55 ± 0.03 ^b,d^	−34	HIS 90 (CN, 2.01)	
**4g**	CN	OCH_3_	9.62 ± 0.03 ^c^	−35	HIS 90 (CN, 2.07)	
**IND**			6.84 ± 0.03 ^b,c^	−49		ARG 120 (anion–cation)
**CLB**			0.05 ± 0.03 ^b^	−43	HIS 90 (SO_2_, 3.05) PHE 158 (SO_2_, 2.92) GLN 192 (NH_2_, 2.69) LEU 352 (NH_2_, 1.95) ARG120 (CF_3_, 2.38)	ARG 120 (pi–cation) PHE 158 (pi–sulfur) ALA 527 (pi–amide)

^a^ IC_50_ value is the compound concentration required to produce 50% inhibition of COX-2 enzyme, expressed as means of three experimental determinations. IND: indomethacin; CLB: celecoxib. ^a–d^ Test compounds not sharing a letter differ significantly (*p* < 0.05).

**Table 5:** The predicted pharmacokinetic properties of all the synthesized compounds.

Compound ID	4a	4b	4c	4d	4e	4f	4g
Rotatable bonds	5	5	5	5	5	5	6
Molecular weight	369.41	387.4	403.86	448.31	405.4	459.29	410.42
H-bond acceptors	3	4	3	3	5	4	5
H-bond donors	0	0	0	0	0	0	0
MLOGP	3.43	3.8	3.91	4.01	1.86	3.11	2.2
GI absorption	High	High	High	High	High	High	High
BBB permeant	Yes	Yes	Yes	Yes	No	No	No
P-gp substrate	No	No	No	No	No	No	No
Lipinski violations	0	0	0	0	0	0	0

**Table 6:** The predicted toxic effects of all the synthesized compounds.

Compound ID	4a	4b	4c	4d	4e	4f	4g
Mutagenic	none	none	none	none	none	none	none
Tumorigenic	none	none	none	none	none	none	none
Reproductive Effective	none	none	none	none	none	none	none
Irritant	none	none	none	none	none	none	none
CYP1A2 inhibitor	Yes	Yes	Yes	Yes	Yes	No	Yes
CYP2D6 inhibitor	No	No	No	No	No	No	No
CYP3A4 inhibitor	No	No	No	No	Yes	Yes	No

## Supplementary Materials

The following are available online at https://www.mdpi.com/2218-273X/9/11/661/s1, Figure S1: FT-IR of methyl 3-benzoyl-7-methyl-2-phenylindolizine-1-carboxylate (**4a**), Figure S2: ^1^H-NMR of methyl 3-benzoyl-7-methyl-2-phenylindolizine-1-carboxylate (**4a**), Figure S3: ^13^C-NMR of methyl 3-benzoyl-7-methyl-2-phenylindolizine-1-carboxylate (**4a**), Figure S4: FT-IR of methyl 3-(4-fluorobenzoyl)-7-methyl-2-phenylindolizine-1-carboxylate (**4b**), Figure S5: ^1^H-NMR of methyl 3-(4-fluorobenzoyl)-7-methyl-2-phenylindolizine-1-carboxylate (**4b**), Figure S6: ^13^C-NMR of methyl 3-(4-fluorobenzoyl)-7-methyl-2-phenylindolizine-1-carboxylate (**4b**), Figure S7: FT-IR of methyl 3-(4-chlorobenzoyl)-7-methyl-2-phenylindolizine-1-carboxylate (**4c**), Figure S8: ^1^H-NMR of methyl 3-(4-chlorobenzoyl)-7-methyl-2-phenylindolizine-1-carboxylate (**4c**), Figure S9: ^13^C-NMR of methyl 3-(4-chlorobenzoyl)-7-methyl-2-phenylindolizine-1-carboxylate (**4c**), Figure S10: FT-IR of methyl 3-(4-bromobenzoyl)-7-methyl-2-phenylindolizine-1-carboxylate (**4d**), Figure S11: ^1^H-NMR of methyl 3-(4-bromobenzoyl)-7-methyl-2-phenylindolizine-1-carboxylate (**4d**), Figure S12: ^13^C-NMR of methyl 3-(4-bromobenzoyl)-7-methyl-2-phenylindolizine-1-carboxylate (**4d**), Figure S13: FT-IR of methyl 7-cyano-3-(4-cyanobenzoyl)-2-phenylindolizine-1-carboxylate (**4e**), Figure S14: ^1^H-NMR of methyl 7-cyano-3-(4-cyanobenzoyl)-2-phenylindolizine-1-carboxylate (**4e**), Figure S15: ^13^C-NMR of methyl 7-cyano-3-(4-cyanobenzoyl)-2-phenylindolizine-1-carboxylate (**4e**), Figure S16: FT-IR of methyl 3-(4-bromobenzoyl)-7-cyano-2-phenylindolizine-1-carboxylate (**4f**), Figure S17: ^1^H-NMR of methyl 3-(4-bromobenzoyl)-7-cyano-2-phenylindolizine-1-carboxylate (**4f**), Figure S18: ^13^C-NMR of methyl 3-(4-bromobenzoyl)-7-cyano-2-phenylindolizine-1-carboxylate (**4f**), Figure S19: FT-IR of methyl 7-cyano-3-(4-methoxybenzoyl)-2-phenylindolizine-1-carboxylate (**4g**), Figure S20: ^1^H-NMR of methyl 7-cyano-3-(4-methoxybenzoyl)-2-phenylindolizine-1-carboxylate (**4g**), Figure S21: ^13^C-NMR of methyl 7-cyano-3-(4-methoxybenzoyl)-2-phenylindolizine-1-carboxylate (**4g**).

## References

[1] Lipscomb J. (2019). Management of Nonsteroidal Anti-inflammatory Drug-Induced Hypersensitivity Reactions. US Pharm..

[2] Ricciotti E., Tang S.-Y., Barekat K., Veglia F., Maseda D., Bittinger K., Bushman F., FitzGerald G.A. (2019). The impact of cyclooxygenase-2 selective and non-isoform selective NSAIDs on the gut microbiota. FASEB J..

[3] Moro M.G., Oliveira M.D.d.S., Oliveira L.R.d., Teixeira S.A., Muscará M.N., Spolidorio L.C., Holzhausen M. (2019). Effects of Selective Versus Non-Selective COX-2 Inhibition on Experimental Periodontitis. Braz. Dent. J..

[4] Takeuchi K., Amagase K. (2018). Roles of cyclooxygenase, prostaglandin E2 and EP receptors in mucosal protection and ulcer healing in the gastrointestinal tract. Curr. Pharm. Des..

[5] Pannunzio A., Coluccia M. (2018). Cyclooxygenase-1 (COX-1) and COX-1 inhibitors in cancer: A review of oncology and medicinal chemistry literature. Pharmaceuticals.

[6] Mirshafiey A., Mortazavi-Jahromi S.S., Taeb M., Cuzzocrea S., Esposito E. (2018). Evaluation of the effect of α-L-guluronic acid (G2013) on COX-1, COX-2 activity and gene expression for introducing this drug as a novel NSAID with immunomodulatory property. Recent Pat. Inflamm. Allergy Drug Discov..

[7] Zarghi A., Zebardast T., Daraie B., Hedayati M. (2009). Design and synthesis of new 1, 3-benzthiazinan-4-one derivatives as selective cyclooxygenase (COX-2) inhibitors. Bioorg. Med. Chem..

[8] Omar Y.M., Abdu-Allah H.H., Abdel-Moty S.G. (2018). Synthesis, biological evaluation and docking study of 1, 3, 4-thiadiazole-thiazolidinone hybrids as anti-inflammatory agents with dual inhibition of COX-2 and 15-LOX. Bioorg. Chem..

[9] Gubin J., de Vogelaer H., Inion H., Houben C., Lucchetti J., Mahaux J., Rosseels G., Peiren M., Clinet M. (1993). Novel heterocyclic analogs of the new potent class of calcium entry blockers: 1-[[4-(aminoalkoxy) phenyl] sulfonyl] indolizines. J. Med. Chem..

[10] Hagishita S., Yamada M., Shirahase K., Okada T., Murakami Y., Ito Y., Matsuura T., Wada M., Kato T., Ueno M. (1996). Potent inhibitors of secretory phospholipase A2: Synthesis and inhibitory activities of indolizine and indene derivatives. J. Med. Chem..

[11] Chaniyara R., Tala S., Chen C.-W., Zang X., Kakadiya R., Lin L.-F., Chen C.-H., Chien S.-I., Chou T.-C., Tsai T.-H. (2013). Novel antitumor indolizino [6, 7-b] indoles with multiple modes of action: DNA cross-linking and topoisomerase I and II inhibition. J. Med. Chem..

[12] Artico M., Massa S., Stefancich G., Silvestri R., Di Santo R., Corelli F. (1989). Potential antitumor agents. III. Synthesis of pyrazolo [3, 4-e] pyrrolo [3, 4-g] indolizine and 1H-pyrazolo [3, 4-e] indolizine derivatives. J. Heterocycl. Chem..

[13] Shrivastava S.K., Srivastava P., Bandresh R., Tripathi P.N., Tripathi A. (2017). Design, synthesis, and biological evaluation of some novel indolizine derivatives as dual cyclooxygenase and lipoxygenase inhibitor for anti-inflammatory activity. Bioorg. Med. Chem..

[14] Chandrashekharappa S., Venugopala K.N., Tratrat C., Mahomoodally F.M., Aldhubiab B.E., Haroun M., Venugopala R., Mohan M.K., Kulkarni R.S., Attimarad M.V. (2018). Efficient synthesis and characterization of novel indolizines: Exploration of in vitro COX-2 inhibitory activity and molecular modelling studies. New J. Chem..

[15] Sandeep C., Venugopala K., Khedr M., Padmashali B., Kulkarni R., Rashmi V., Odhav B. (2017). Design and synthesis of novel indolizine analogues as COX-2 inhibitors: Computational perspective and in vitro screening. Indian J. Pharm. Educ. Res..

[16] (2006). SAINT Version 7.60a.

[17] Sheldrick G.M. (1997). SHELXS-97, SHELXL-2014 and SADABS Version 2.05.

[18] Barbour L.J. (2001). X-Seed—A Software Tool for Supramolecular Crystallography.

[19] Jerry L.A., Leonard J.B. (2003). Molecular Graphics: From Science to Art. Cryst. Growth Des..

[20] Barbour L. (1999). X-Seed, Graphical Interface to SHELX-97 and POV-Ray.

[21] Daina A., Michielin O., Zoete V. (2017). SwissADME: A free web tool to evaluate pharmacokinetics, drug-likeness and medicinal chemistry friendliness of small molecules. Sci. Rep..

[22] Pollastri M.P. (2010). Overview on the Rule of Five. Curr. Protoc. Pharmacol..

[23] Yu D.K. (1999). The contribution of P-glycoprotein to pharmacokinetic drug-drug interactions. J. Clin. Pharmacol..

[24] Fromm M. (2003). Importance of P-glycoprotein for drug disposition in humans. Eur. J. Clin. Invest..

[25] Sander T., Freyss J., von Korff M., Rufener C. (2015). DataWarrior: An open-source program for chemistry aware data visualization and analysis. J. Chem. Inf. Model..

[26] Lin J.H. (2006). CYP induction-mediated drug interactions: In vitro assessment and clinical implications. Pharm. Res..

[27] https://www.Ccdc.Cam.Ac.Uk.

[28] Farrugia L. (1999). WinGX suite for small-molecule single-crystal crystallography. J. Appl. Crystallogr..

[29] Macrae C.F., Bruno I.J., Chisholm J.A., Edgington P.R., McCabe P., Pidcock E., Rodriguez-Monge L., Taylor R., van de Streek J., Wood P.A. (2008). Mercury CSD 2.0—New features for the visualization and investigation of crystal structures. J. Appl. Crystallogr..

[30] Kalgutkar A.S., Crews B.C., Rowlinson S.W., Marnett A.B., Kozak K.R., Remmel R.P., Marnett L.J. (2000). Biochemically based design of cyclooxygenase-2 (COX-2) inhibitors: Facile conversion of nonsteroidal antiinflammatory drugs to potent and highly selective COX-2 inhibitors. Proc. Natl. Acad. Sci. USA.

[31] Bhattacharyya D.K., Lecomte M., Rieke C.J., Garavito R.M., Smith W.L. (1996). Involvement of arginine 120, glutamate 524, and tyrosine 355 in the binding of arachidonate and 2-phenylpropionic acid inhibitors to the cyclooxygenase active site of ovine prostaglandin endoperoxide H synthase-1. J. Biol. Chem..

[32] Greig G.M., Francis D.A., Falgueyret J.-P., Ouellet M., Percival M.D., Roy P., Bayly C., Mancini J.A., O’Neill G.P. (1997). The interaction of arginine 106 of human prostaglandin G/H synthase-2 with inhibitors is not a universal component of inhibition mediated by nonsteroidal anti-inflammatory drugs. Mol. Pharmacol..

[33] Lombardo F., Gifford E., Shalaeva M.Y. (2003). In silico ADME prediction: Data, models, facts and myths. Mini Rev. Med. Chem..

[34] Smith P., Sorich M., Low L., McKinnon R., Miners J. (2004). Towards integrated ADME prediction: Past, present and future directions for modelling metabolism by UDP-glucuronosyltransferases. J. Mol. Graph. Model..

[35] Veber D.F., Johnson S.R., Cheng H.-Y., Smith B.R., Ward K.W., Kopple K.D. (2002). Molecular properties that influence the oral bioavailability of drug candidates. J. Med. Chem..

